# Submonolayer Uniformity of Type II InAs/GaInSb W-shaped Quantum Wells Probed by Full-Wafer Photoluminescence Mapping in the Mid-infrared Spectral Range

**DOI:** 10.1186/s11671-015-1104-z

**Published:** 2015-10-15

**Authors:** Mateusz Dyksik, Marcin Motyka, Grzegorz Sęk, Jan Misiewicz, Matthias Dallner, Robert Weih, Martin Kamp, Sven Höfling

**Affiliations:** Laboratory for Optical Spectroscopy of Nanostructures, Department of Experimental Physics, Wrocław University of Technology, St. Wybrzeże Wyspiańskiego 27, Wrocław, 50-370 Poland; Technische Physik, Wilhelm Conrad Röntgen Research Center for Complex Material Systems, University of Würzburg, Am Hubland, Würzburg, 97074 Germany; School of Physics and Astronomy, University of St. Andrews, North Haugh, St. Andrews, KY16 9SS UK

**Keywords:** Spatially resolved photoluminescence, Type II quantum wells, Mid-infrared, Fourier transform spectroscopy, Interband cascade lasers

## Abstract

The spatial uniformity of GaSb- and InAs substrate-based structures containing type II quantum wells was probed by means of large-scale photoluminescence (PL) mapping realized utilizing a Fourier transform infrared spectrometer. The active region was designed and grown in a form of a W-shaped structure with InAs and GaInSb layers for confinement of electrons and holes, respectively. The PL spectra were recorded over the entire 2-in. wafers, and the parameters extracted from each spectrum, such as PL peak energy position, its linewidth and integrated intensity, were collected in a form of two-dimensional spatial maps. Throughout the analysis of these maps, the wafers’ homogeneity and precision of the growth procedure were investigated. A very small variation of PL peak energy over the wafer indicates InAs quantum well width fluctuation of only a fraction of a monolayer and hence extraordinary thickness accuracy, a conclusion further supported by high uniformity of both the emission intensity and PL linewidth.

## Background

Photoluminescence (PL) imaging has been widely employed as a qualitative measurement method of semiconductor structures. As a consequence of its high sensitivity to various optical singularities, investigation of the sample’s interface quality is possible in the case of undesirable localized states present near or at the particular surfaces. Here, the near-field photoluminescence spectroscopy is at forefront, providing spatial resolution at submicronic scale in the near-infrared spectral range [1, 2]. More conventional PL mapping has also been found to be useful in examining the exciton diffusion in quantum wells [3], investigating the doping and structural composition of semiconductor heterostructures [4] and studying the emission properties of microcavity devices [5]. On the other hand, the absorption-like experiments, including contactless electroreflectance [6] and optical absorption measurements [7], have also been successfully applied to probe inhomogeneities. In addition, the methods of optical spectroscopy also found their application in industrial testing, where fast and non-destructive wafer characterization is crucial. There, the PL mapping is the most common and developed technique including large-scale systems capable of mapping 300-mm wafers [8].

The abovementioned measurements are typically performed in a near-infrared set-up configuration with a monochromator as a dispersive element and a charge-coupled device or a photodiode as a detector. There are, however, just a few reports on mapping in the mid-infrared spectral range utilizing a Fourier transformed infrared (FTIR) spectrometer, regarding mainly the uniformity investigations of mercury cadmium telluride epilayers [9, 10]. No spatially resolved PL study of quasi-two-dimensional structures emitting in this spectral range has been reported yet, which would be of high importance if the fabrication of devices, such as semiconductor lasers, is based on these systems.

Recently, nanostructures with broken gap alignment have attracted a lot of interest as a promising configuration for active regions of both lasers [11–13] and detectors [14, 15], operating in the mid-infrared region, even beyond 10-μm. Concerning laser sources, R. Q. Yang in 1995 presented the basic concept of employing type II quantum wells (QWs) into a previously presented cascade scheme, resulting in an interband cascade laser (ICL) [16]. The natural candidate for the active region of ICLs is the so-called W-shaped type II quantum well made of InAs and GaInSb layers to confine electrons and holes, respectively [16]. Such a type-II-based approach provides the effective band gap reduction (an indirect transition in the real space) and reduces non-radiative processes, such as Auger recombination [17]. The state-of-the-art ICLs based on GaSb-substrate have already reached suitable operating parameters [18] allowing for their application in gas sensing devices [19]. Another approach, developed in parallel, is to employ InAs substrates, which has also reached some important milestones, e.g. the only ICLs emitting beyond 6 μm at room temperature [20] have been grown on InAs substrates, with the record long wavelength emission at around 10 μm [21] operating in pulsed mode.

The region of mid-infrared is full of absorption lines of environmentally relevant gasses, like hydrocarbons CO, CO_2_, N_2_O and NH_3_, to name a few. The utilization of ICLs as a laser source has been already reported [22] in sensing systems for formaldehyde [23], acetylene impurities in ethylene and polyethylene [24] and nitric oxide [25].

As mentioned above, the active region of a typical ICL is based on a series of “W”-shaped type II quantum wells connected by electron and hole injectors. It has already been discussed elsewhere [26, 27] that the width of the InAs layer in these QWs is strongly correlated with the emitted wavelength, whilst the variation of the GaInSb layer thickness usually has a weaker impact on the transition energy. The width of both of these layers in a typical ICL is of the order of a few nanometres. Depending on the structure details, the change of the InAs layer thickness by approximately one monolayer (ML), (0.3 nm) in the spectral region around 3 μm shifts the emission wavelength by about 0.5 μm, whereas in the further infrared region, around 6 μm, which usually means using thicker InAs layers. The same procedure (the change of the InAs layer thickness by about 0.3 nm) leads to the shift of emission by 2 μm already. This thickness sensitivity makes the fabrication in the larger scale challenging, because the layer thickness fluctuations over the wafer by one ML can make the structure completely unsuitable as a laser for a given gas sensing application. Therefore, both the ultra-high growth accuracy and the uniformity testing methodology are necessary for developing nowadays devices of that kind.

The aim of this work is to examine the spatial homogeneity of emission (the PL peak spectral position, linewidth understood as the peak full width at half maximum (FWHM) and PL intensity) in type II quantum wells by measuring mid-infrared photoluminescence maps. Two 2-in. wafers containing the type II AlSb/InAs/GaInSb/InAs/AlSb quantum wells grown on the GaSb and InAs substrates have been scanned. The data obtained for both wafers are analysed and compared in order to estimate which fraction of the wafer is sufficiently uniform to be further processed into a fully operational ICL device.

## Methods

The studied structures have been grown by a solid-source Molecular Beam Epitaxy system equipped with valved cracker cells for both antimony and arsenic. Two 2-in. wafers on (100) oriented InAs and GaSb substrates containing the type II quantum wells have been investigated. The active region has been designed in the form of the common “W-like” scheme, repeated five times for both samples. The “W”-shaped active part of the GaSb-based structure, optimized for emission at 3.3 μm, consists of two 1.5-nm-wide InAs layers confining the electrons and a 3.5-nm-wide Ga_0.65_In_0.35_Sb layer in between for the hole confinement. These layers are surrounded by 2.5-nm-thick AlSb barriers. Each “W”-shaped QW is separated by a 20-nm-thick GaSb layer. The InAs-based structure, aiming at a longer wavelength emission (around 6 μm), has a slightly different sample layout: each active region is separated by a 25-nm-thick lattice-matched GaAsSb layer, and an InAs layer for electron confinement has a thickness of 2.95 nm, whereas a 3-nm-wide Ga_0.76_In_0.24_Sb layer confines holes.

The spatially resolved photoluminescence studies have been performed with a Bruker Fourier transform infrared spectrometer Vertex 80v operating in the step-scan mode. Both liquid nitrogen cooled InSb and mercury cadmium telluride (MCT) photo-detectors were used for the PL mapping of the GaSb- and InAs-based structures, respectively. An external pump beam provided by a 660-nm semiconductor laser diode was mechanically chopped at a frequency of 275 Hz. This allows for a phase sensitive detection of the optical response using a lock-in amplifier. For more information about the measurement set-up, the reader is referred to [28, 29].

In order to map the photoluminescence response in the scale of the full 2-in. wafer, the samples were mounted on an x-y stage. The spatial resolution was defined by the pump beam diameter, which was focused on the sample to a spot of 0.5 mm^2^. In total, about 360 PL spectra were collected for each wafer. Assuming it takes 5 min to capture one PL response, 30 h were required to spatially cover one wafer. Each spectrum gives a contribution to a circular mesh of a constant step, which reproduces the sample’s dimensions.

## Results and Discussion

**Fig. 1:**
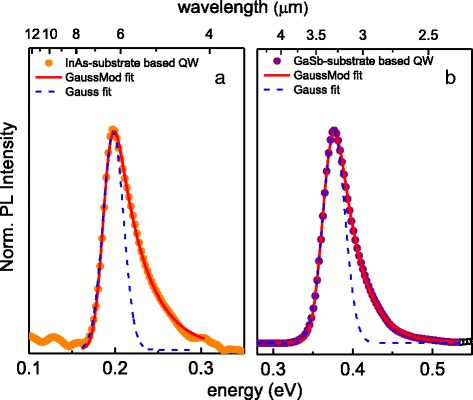
Room temperature PL spectra from **a** InAs-based and **b** GaSb-based structures. The *red solid line* stands for a fit by an exponentially modified Gaussian. For comparison, a symmetric Gaussian function is plotted in *dashed blue*

Figure 1a, b present examples of room temperature PL spectra collected from the type II AlSb/InAs/GaInSb/InAs/AlSb quantum wells grown on the InAs and GaSb substrates, respectively. The observed PL linewidth for both is about 40–60 meV, being of the order of thermally broadened line of 2 k_B_T width. The PL response, with a hot carrier emission contribution resulting in the high energy tail of the spectra, has been fitted by the exponentially modified Gaussian function—a convolution of an exponential decay originating from the k_B_T tail and the symmetric Gaussian function. The PL peak parameters to be discussed throughout this publication, energetic positions, linewidths (FWHM) and integrated PL intensities, are evaluated from the analysis of such fitting curves (see the red fitting lines in Fig. 1). For comparison, the symmetric Gaussian function has been shown in Fig. 1, which demonstrates the significance of including the exponentially modified Gaussian into the analysis.

**Fig. 2:**
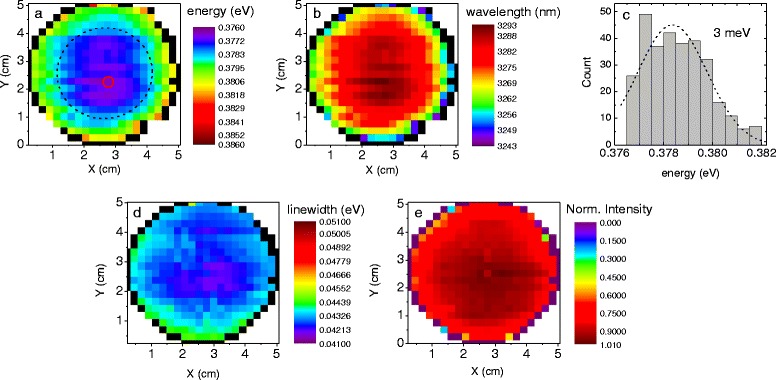
Data extracted from PL measurements of the GaSb-based structure. **a** Spatially resolved PL peak energy map with the *dashed black line* indicating a region of the highest uniformity and the *tiny dashed red circle* corresponding to the grid point of minimum energy. **b** A corresponding map of emitted wavelength and **c** PL peak energy histogram. The **d** linewidth (FWHM) and **e** normalized integral intensity distribution are also presented

The spatially resolved map of the PL peak energy position (the transition between the first electron and the first heavy hole confined states) in the GaSb-based structure is presented in Fig. 2a. It shows a very high uniformity of the wafer in the sense of the fundamental type II transition—*E*_e1hh1_. The emission energy divergence between the wafer’s centre and its periphery equals to ~4 meV, relating to the InAs thickness variation of about 0.1 ML (see the examples given above). For an easy direct comparison, a related emission map in the wavelength scale is show in Fig. 2b, exhibiting an “along the radius” fluctuation of only about 30 nm in this particular spectral range. Several processes may contribute to the energy emission discrepancy: the layers thickness and/or composition variation due to radial inhomogeneity of the corresponding atomic beams or substrate temperature during the epitaxial growth. It has been shown before [26, 27] and mentioned above that the emission wavelength of the discussed QW is very sensitive to the InAs thickness, whilst the GaInSb width fluctuations have a minor impact on the transition energy. The fluctuation of the indium content in the ternary GaInSb layers across the sample is linked to the InAs thickness fluctuation and also affects the energy of the discussed transition between the confined states in the QW, which cannot be excluded in the final discussion.

The histogram in Fig. 2c presents the distribution of the *E*_e1hh1_ energy of the GaSb-based structure extracted from the X-Y spatially resolved map (Fig. 2a). It exhibits a normal distribution plotted with the dashed line, with an FWHM of 3 meV. The maximum number of counts is recorded for the energy of 0.377 eV. Together with the bars representing energies of 0.376 and 0.378 eV, it fills the region marked with the dashed circle in Fig. 2a. In this particular region, the emission energy remains nearly constant with a variation of 1 meV. One can notice a similar region in Fig. 2b, with the wavelength emission fluctuations of 8 nm only.

Figure 2d, e exhibit a similar trend and uniformity level for the linewidth and the normalized integral intensity, respectively. The former one presents the linewidth distribution over the entire structure with the minimum values concentrated in the wafer’s centre. The narrowest linewidth indicated by the shades of blue in Fig. 2d also fills a similar area as the one indicated by the dashed circle in Fig. 2a, spreading a bit to the top of the sample. Such high uniformity of the linewidth over the entire wafer is actually of the order of a standard error of an individual measurement, which will be discussed below. The linewidth is also distorted by the high-energy tail, indicating that the spectral broadening results mostly from the thermal broadening.

The normalized integral intensity for the GaSb-based wafer is depicted in Fig. 2e. A moderate trend towards lower values is visible, with a drop in the intensity of 25 % when moving to outlying grid points, which might be less important in the case of processing into a fully operational device, as long as the peak wavelength is well kept.

The maps in Fig. 2 are affected by an outer ring in black indicating a region where the QW did not form due to shading from the wafer holder. This area is characterized by the lack of signal resulting from the radiative recombination between the QW confined states (only the fundamental energy gap of the GaSb substrate material has been recorded).

**Fig. 3:**
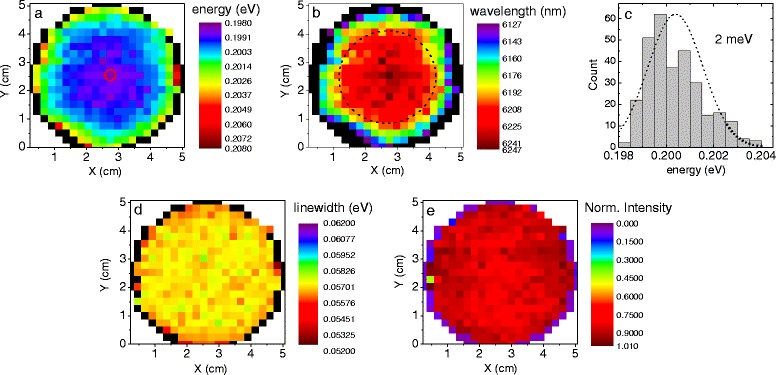
Data extracted from PL measurements of the InAs-based structure. **a** Spatially resolved PL peak energy map and **b** corresponding wavelength distribution. The *dashed circle* represents the region of ultra-high uniformity. **c** The PL peak energy histogram. The **d** linewidth (FWHM) and **e** normalized integral intensity distribution are also presented

Similar results have been obtained for the second wafer containing the type II quantum wells grown on the InAs substrate designed for the emission around 6.2 μm. The *E*_e1hh1_ PL map shown in Fig. 3a reveals regularity as in the case of the previously discussed structure. Analogous to Fig. 2a, the energy distribution in the case of the InAs-based structure is also highly uniform in the scale of the full 2-in. wafer, with the lowest value of 0.198 eV lying in the very centre and varying by ~4 meV to its periphery. Though the same value has been estimated for the GaSb-based structure, now the spectral region for this sample determines an enhanced wavelength variation. This is presented in Fig. 3b and equals to the change in the emission wavelength of ~60 nm. One can also notice a similar region with ultra-high regularity marked with the dashed circle. Within this region, the wavelength fluctuations have been estimated to be 20 nm. Both Figs. 2a and 3a are plotted in the same scale in order to give proper guidance to the eye.

Although the mentioned wavelength irregularities of the order of 60 nm in the case of the InAs-based structure might be considered as significant with respect to some applications, it has been already presented [30] that the emission energy of an operating device can be electrically tuned by at least that amount. This allows for these minor growth inaccuracies to be then fully compensated by an external electric field in an operating ICL.

Figure 3c presents the histogram of data extracted from the spatially resolved *E*_e1hh1_ map. It reveals a normal distribution with an FWHM of 2 meV and is consistent with the previously discussed histogram for the GaSb-based sample. In order to estimate the standard error of an individual measurement of E_e1hh1_, the linewidths and integral intensities, the PL signal has been measured ten times for a selected grid point, marked with a tiny red circle in Figs. 2a and 3a, for the GaSb- and InAs-based samples, respectively. It is worth mentioning that the estimated overall standard error resulting from the spectrometer inaccuracy, the measurement conditions and the fitting procedure is of the order of 1 meV. This indicates that the previously mentioned energy and linewidth fluctuations of 1 meV in the region of ultra-high uniformity marked with the dashed circle in Fig. 2a result rather from the experimental spectrometer inaccuracies than from the interface roughness, layer fluctuations or composition fluctuations in that part of the samples.

Figure 3d, e present the linewidth and normalized integral intensity maps, respectively. The former one for the InAs-based structure exhibits a practically constant value of 57 meV over the entire structure. This is, to some extent, a more homogeneous linewidth behaviour than for the GaSb-based structure, manifested also by the normalized integral intensity map depicted in Fig. 3e. The InAs wafer exhibits nearly isotropic behaviour with the maximum intensities in the rim’s vicinity.

**Fig. 4:**
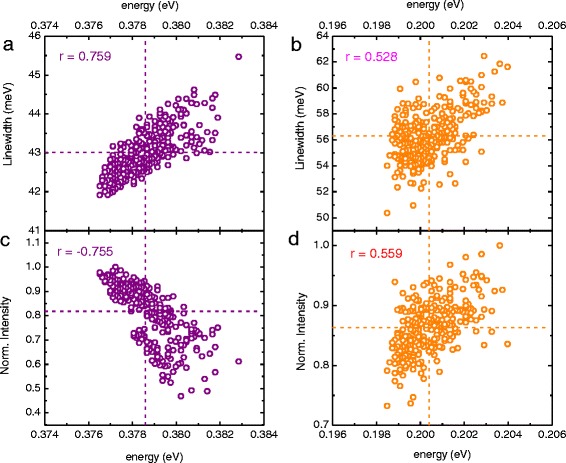
The correlation plots regarding emission energy, intensity and linewidth. *Purple* and *orange open symbols* correspond to GaSb- and InAs-based structures, respectively. *r* stand for the Pearson product-momentum correlation coefficient. The vertical and horizontal lines denote the mean values of corresponding quantities

In addition, we have investigated the correlation of the PL peak energy with both the integral intensity and linewidth. As previously shown for the energy and linewidth maps for the GaSb-based structure (Fig. 2a, d), the lowest measured PL energy concentrates in the wafer’s centre and correlates with the lowest broadening observed in the same region. This situation is illustrated in Fig. 4a, showing a correlation plot of the linewidth vs. the PL peak energy. The strong correspondence is observed between these two values and estimated by means of the Pearson product-momentum correlation coefficient *r*, which can be expressed as

$$ r=\frac{\mathrm{cov}\left(x,y\right)}{\sigma_x\;{\sigma}_y}=\frac{\mu_{xy}-{\mu}_x{\mu}_y}{\sigma_x\;{\sigma}_y}, $$

where cov(*x*,*y*) is the covariance of *x* and *y* variables and *μ*_*x*_, *μ*_*y*_ and *μ*_*xy*_ represent their averages and product, respectively. The *σ*_*x*_ and *σ*_*y*_ correspond to standard deviations. For the PL peak energy and linewidth, *r* = 0.759 and *r* = 0.528 were found in the case of the GaSb- and InAs-based sample, respectively. The correlation plot of the latter is presented in Fig. 4b. Both indicate a very strong correlation, with the purple and orange open circles representing a similar pattern, exhibiting a moderate tendency towards an increment in broadening with the blue shift of the PL peak energy. For both figures, the dashed horizontal and vertical lines denote the mean values of linewidth and *E*_e1hh1_ energy, respectively. Consequently, the same calculation for the PL peak energy and the normalized integral intensity revealed that *r* = −0.755 and *r* = 0.559 for the GaSb- and InAs-based structures. The calculated Pearson coefficients agree well with the correlation plots shown in Fig. 4c, d, respectively. The former one is a well-known situation, as the normalized integral intensity decreases with the increasing peak energy: an equivalent of movement along the wafer’s radius—starting in its centre with the lowest energy and the highest integral intensity. On the contrary, the PL peak energy—normalized integral intensity correlation plot for the InAs-based structure (Fig. 4d) exhibits a different behaviour. One can notice in Fig. 3a an outer ring formed by grid points in green related to a higher PL peak energy. This outer ring is correlated with a similar region in Fig. 3e, representing the maximum values of the measured intensity.

The determined Pearson coefficients for the InAs-based sample are almost 25 % smaller than in the case of the GaSb-based structure, indicating a rather weak correlation. This is also visible by an almost-vanishing linearity in Fig. 4b, d. However, the weak correlation or its lack might be considered beneficial in the case of processing a wafer into a fully operational device—the parameter’s inhomogeneity, resulting from the complex growth procedure, uniformly influences the sample.

## Conclusions

The large-scale mid-infrared photoluminescence studies have been performed on the GaSb- and InAs substrate-based structures in order to present systematic examination of spatially resolved structural properties. For the first time, a Fourier transform infrared spectrometer has been employed in order to study the spatially resolved emission properties of low-dimensional structures like the type II InAs/GaInSb W-shaped quantum wells. The photoluminescence spectra in the number of a few hundred per wafer were captured and analysed in the terms of the emission wavelength, linewidth and PL peak intensity variations. Both the GaSb- and InAs-based structures exhibited ultra-high wavelength emission stability. The analysis of the spatially resolved maps of the PL peak linewidth and the normalized integral intensity for both samples revealed that almost the entire wafer’s area meets the design criteria and can be used for further processing, if included into a full device structure.

## Abbreviations

FTIR: Fourier transform infrared spectroscopy
FWHM: full width at half maximum
ICL: interband cascade laser
MBE: molecular beam epitaxy
MCT: mercury cadmium telluride
PL: photoluminescence
QWs: quantum wells
